# A Novel Peptide from Abalone (*Haliotis discus hannai*) to Suppress Metastasis and Vasculogenic Mimicry of Tumor Cells and Enhance Anti-Tumor Effect In Vitro

**DOI:** 10.3390/md17040244

**Published:** 2019-04-24

**Authors:** Fang Gong, Mei-Fang Chen, Yuan-Yuan Zhang, Cheng-Yong Li, Chun-Xia Zhou, Peng-Zhi Hong, Sheng-Li Sun, Zhong-Ji Qian

**Affiliations:** 1 College of Food Science and Technology, Guangdong Ocean University, Zhanjiang 524088, China; m13025612271@163.com (F.G.); meifangchen93@163.com (M.-F.C.); zyyla92@126.com (Y.-Y.Z.); chunxia.zhou@163.com (C.-X.Z.); hongpengzhigdou@163.com (P.-Z.H.); 2 School of Chemistry and Environment, Guangdong Ocean University, Zhanjiang 524088, China; cyli_ocean@163.com (C.-Y.L.); xinglsun@126.com (S.-L.S.); 3 Shenzhen Institute of Guangdong Ocean University, Shenzhen 518114, China

**Keywords:** abalone, peptide, vasculogenic mimicry, metastasis, MMPs, HIF-1α

## Abstract

Vasculogenic mimicry (VM) formed by tumor cells plays a vital role in the progress of tumor, because it provides nutrition for tumor cells and takes away the metabolites. Therefore, the inhibition of VM is crucial to the clinical treatment of tumors. In this study, we investigated the anti-tumor effect of a novel peptide, KVEPQDPSEW (AATP), isolated from abalone (*Haliotis discus hannai*) on HT1080 cells by migration, invasion analysis and the mode of action. The results showed that AATP effectively inhibited MMPs by blocking MAPKs and NF-κB pathways, leading to the downregulation of metastasis of tumor cells. Moreover, AATP significantly inhibited VM and pro-angiogenic factors, including VEGF and MMPs by suppression of AKT/mTOR signaling. In addition, molecular docking was used to study the interaction of AATP and HIF-1α, and the results showed that AATP was combined with an active site of HIF-1α by a hydrogen bond. The effect of AATP on anti-metastatic and anti-vascular in HT1080 cells revealed that AATP may be a potential lead compound for treatment of tumors in the future.

## 1. Introduction

A tumor is a mass or lump formed by cells that have unregulated growth potential [1]. For malignant tumors, tumor cells can distribute diffusely and form the second tumor in distal site [2]. Cancer is a serious threat to human life and health. For cancer patients, metastasis of tumor cells affects the function of other tissues, and results in the death of the patient. In addition, the blood vessels provide the nutrition for the tumor cell’s growth in the course of tumorigenesis. Thus, the inhibition of metastasis and angiogenesis of tumor cells is of great significance for clinical treatment of tumors. 

The detachment of tumor cells from their primary site, the degradation of basement and extracellular matrix (ECM), and the formation of tumor vessels are significant parts of the process of tumor cells metastasis [3,4]. The secretion of matrix metalloproteinase (MMP) from tumor cells is relevant to the degradation of basement and ECM that resist metastasis of tumor cells. Previous studies have found that MMP-2 and MMP-9 can degrade type IV collagen, which is an important component of ECM [5]. Thus, inhibition of MMPs is extremely crucial in tumor therapy. 

Because the unregulated growth of tumor cells needs a large amount of nutrition and oxygen, the formation of new blood vessels is necessary, which not only provides ongoing nutrition and oxygen for tumor growth and takes away metabolites at the same time, but also transports tumor cells to target organs and tissue, which provide a necessary condition for tumor metastasis [6]. Therefore, numerous studies have paid attention as to how to inhibit angiogenesis, which is important for anti-tumor. Besides traditional tumor angiogenesis and vasculogenesis is produced by endothelial cells, vasculogenic mimicry (VM) has attracted attention as a novel blood supply. VM is not found in the healthy body, but is unique to tumor tissue, where it can promote cancer progression by the formation of blood vessel-like structures, independent of vascular endothelial cells [7]. It has been found in various aggressive tumors, such as breast cancer, pancreas cancer, liver cancer and various sarcomas [8]. VM is also implicated in poor patient clinical prognoses, because previous studies have focused on the treatment of blood vessels produced by endothelial cells, rather than VM by tumor cells [9]. 

The fast growth of tumor cells generally generates a hypoxic microenvironment within the tumor. Under this reduced state of cellular oxygen availability, the hypoxia-inducible factor (HIF)-1α expression is frequently fiercely elevated [10]. HIF-1α is a major transcription factor that mediates oxygen homeostasis, which is disrupted in disorders affecting the circulatory system and in cancer [11]. Increasing evidence is suggesting that HIF-1α could facilitate tumor growth by disrupting metabolic balance, accelerating angiogenesis, increasing cell survival, inhibiting cell apoptosis, as well as increasing drug resistance [12]. Hypoxic induced factor (HIF-1α) overexpression in tumor cells promotes the expression of vascular endothelial growth factor (VEGF), related to VM formation [13,14]. The level of matrix metalloproteinases (MMPs) and the 5γ2 chain of laminin were overexpressed in highly aggressive tumor cells [15]. That excessive expression of MMPs and the presence of laminin receptor on the surface of tumor cells contributes to cells to adhere to more laminin. The activation of MMPs can facilitate the formation of VM by separating laminin [16]. HT1080 is a fibrosarcoma cell line which has been used widely in biomedical research. The cell line was isolated from tissue taken in a biopsy of a fibrosarcoma present in a 35-year-old human male. The sample supplied by the patient had been not subjected to radio or chemotherapy, making it less likely that unwanted mutations were introduced into the cell line. The cell line carries an IDH1 mutation and an activated N-ras oncogene. HT1080 cells are composed of malignant cells, and generally are used to model metastatic cells because MMPs are overexpressed by PMA and other stimulations. In addition, under hypoxic stimulation, HT1080 cells can generate pro-angiogenic factor, including HIF-1α, VEGF and MMPs to promote tumor angiogenesis, and is an ideal model for studying tumor invasion and metastasis. 

Marine organisms have attracted intense attention as an effective bioactive source, including sponges, macroalgae, microalgae and bacteria. These creatures are usually made into nutraceuticals and pharmaceuticals. Abalone is a marine gastropod feeding on seaweed, and is commonly considered as very precious food in Asian markets. More and more studies have found that abalone consists of many vital moieties like good protein, fatty acid and polysaccharides, which not only provides the basic nutrition for human health, but possesses the ability for anti-microbial, anti-oxidant, anti-thrombotic, anti-inflammatory and anti-cancer [14,15,16,17,18] uses. Qian [19] found that two peptides from abalone can suppress MMP-2 and MMP-9 expressions to inhibit the migration of HT1080 cells. A novel antimicrobial peptide, Ranatuerin-2PLx, showed inhibitory potential in the proliferation of cancer cells [2]. Peptides have attracted more and more attention as bioactive resources, and are wildly applied in clinical trials because of their high bioactivity and easy synthesis.

The research of abalone in anti-cancer treatment mainly focused on the effect of polysaccharides and the crude extract of abalone, however, and there were few studies about abalone polypeptides. In the present study, HT1080 cells serve as a tumor mold, due to its high metastasis ability, to investigate the anti-tumor effect of a peptide AATP from abalone *haliotis discus hannai.*

## 2. Results

### 2.1. Cell Viability and Colony Formation Assay

**Figure 1:**
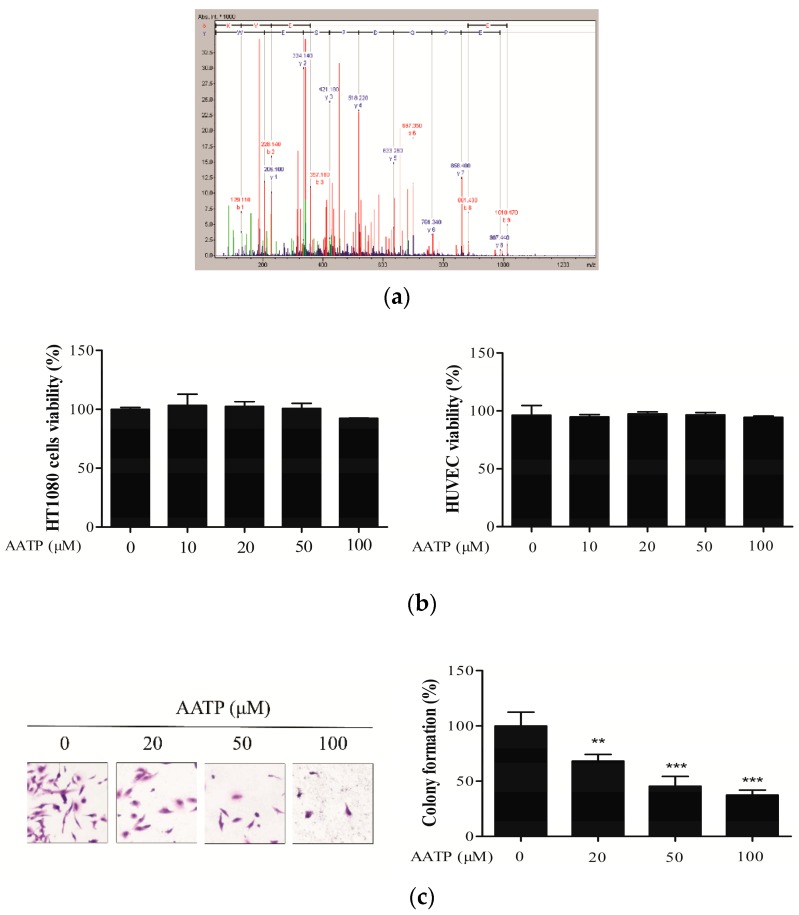
(**a**) Identification of molecular mass and amino acid sequence of Lys-Val-Glu-Pro-Gln-Asp-Pro-Ser-Glu-Trp (AATP). (**b**) Effect of AATP on the viability of HT1080 cells and HUVECs. Cells grown were treated with different concentrations of AATP (10, 20, 50 and 100 μM) for 24 h and relative cell viability was assessed by the MTT assay. (**c**) Anchorage-dependent colony formation in the presence or absence of AATP was visualized by staining with crystal violet solution (*n* = 3 per group). The photographs of tumor cells invasion were taken using inverted microscope at 24 and 48 h and analyzed with ImageJ. ** *p* < 0.01 and *** *p* < 0.001 vs. untreated control.

The peptide AATP (MW = 1214.30 Da) isolated from abalone was obtained according to methods from our previous studies [20]. The amino acid sequence of the purified AATP was determined to be Lys-Val-Asp-Ala-Gln-Asp-Pro-Ser-Glu-Trp (Figure 1a). 

Initially, MTT assay was utilized to assess the cytotoxicity of AATP on HT1080 cells and HUVECs. As shown in Figure 1b, there was no significant difference in cell viability between AATP- treated cells and untreated cells. Thus, all tested concentrations (10, 20, 50 and 100 μM) of AATP have no cytotoxicity on HT1080 cells and HUVECs, which revealed that AATP is non-toxic to tumor cells as well as nontumor cells. Therefore, 10–100 μM of AATP could be used for further experiments. The colony formation assay indicated that the number of colonies of HT1080 cells were sharply reduced by AATP treatment compared with the untreated group (Figure 1c), suggesting AATP exerted inhibitory effect on tumor cells.

### 2.2. AATP Inhibits Tumor Cells Migration and Invasion

**Figure 2:**
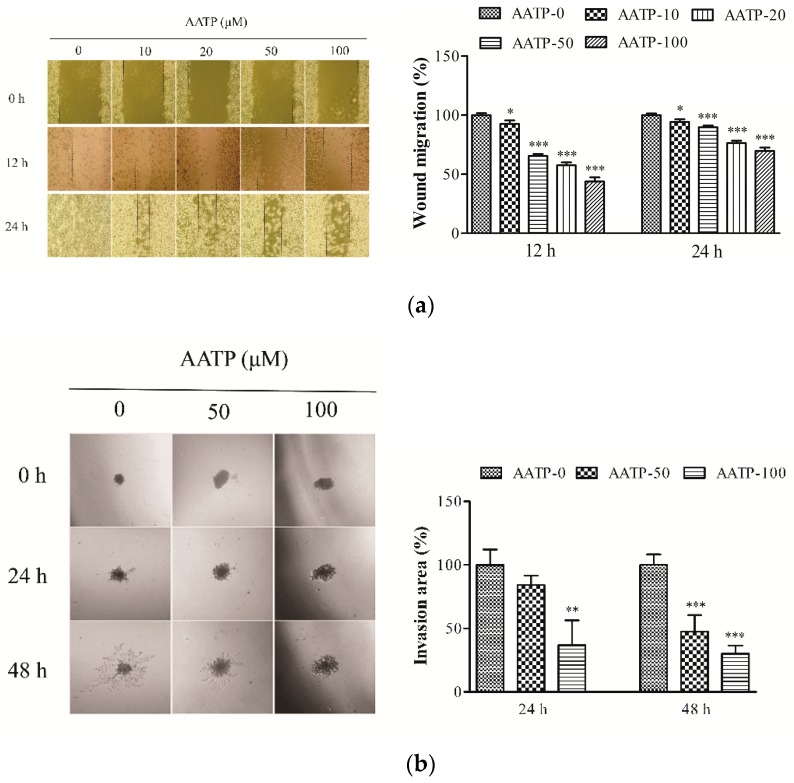
(**a**) Injury lines were made on the confluent cell monolayer, and the effects of AATP on cells migration were monitored for 12 h and 24 h. Cell motility was measured in five selected fields and calculated based on the width of injury at 0 h. (**b**) AATP inhibits cells invasion in 3D sitting. The mixture of cell spheroid combined with Matrigel and type I collagen was seeded on pre-coated Matrigel 48-well plates for 30min, and incubated with a medium containing 50 and 100 μM AATP. The photographs of tumor cells invasion were taken using inverted microscope at 24 and 48 h and analyzed with ImageJ. * *p* < 0.05, ** *p* < 0.01 and *** *p* < 0.001 vs. untreated control.

The migration assay and cancer cell spheroid assay were carried out to estimate the impact of AATP on cancer cells migration and invasion. As exhibited in Figure 2a, compared with control cells, the migration of cells treated by AATP was obviously down-regulated in a dose- and time-dependent manner. Protease secreted by tumor cells can degrade basement and ECM, which contribute to tumor cells metastasis. In Figure 2b, the invasion area of tumor cells treated with 50 and 100 μM AATP was smaller than the control cells, which suggested that AATP treatment effectively inhibits proteolytic activities implicated in degradation of basement and ECM, and suppresses the cell invasion. The results revealed that AATP may be a potential inhibitor for metastatic therapy.

### 2.3. AATP Reduces PMA-induced MMPs Expression and Suppresses Proteolytic Activities in HT1080 Cells

MMPs play an important role in tumor metastasis because MMPs can degrade the surrounding tissue of tumor cells, which creates a place for tumor blood vessels to form. In order to determine the anti-metastatic ability of AATP, we investigated the transcriptional levels of MMPs including MMP-1, -2, -3, -9, -13 as well as activity and protein expression of MMP-2, -9 in HT1080 cells by using Real-Time quantitative reverse transcription-PCR (qPCR), gelatin zymography, and western blotting analysis. 

**Figure 3:**
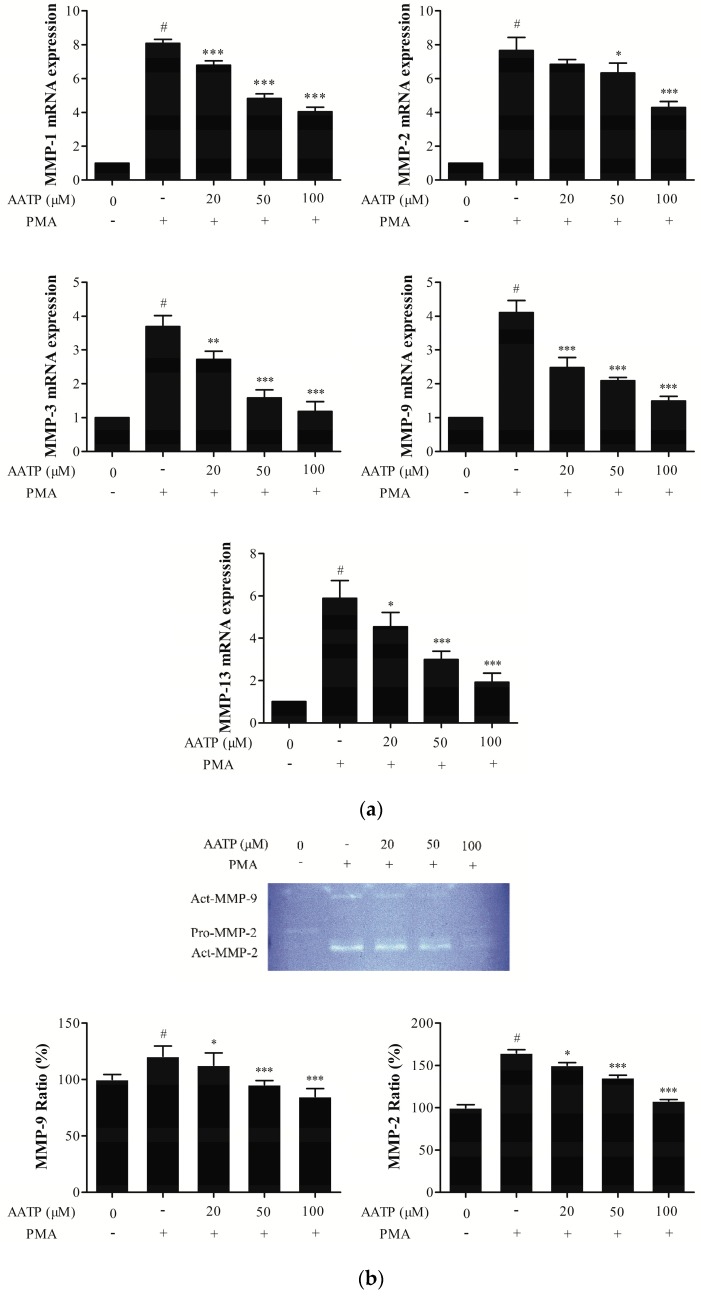

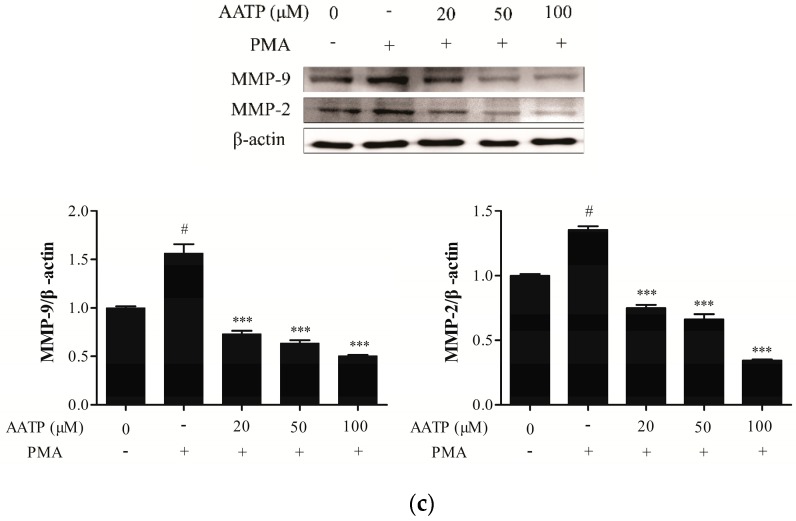
Effect of AATP treatment on expression of matrix metalloproteinase (MMPs) (**a**) Cells treated with AATP for 1 h were incubated in PMA (10 ng/mL). After 24 h, the expression of MMPs RNA was analyzed by quantitative real time PCR. β-actin was used as a loading control. (**b**) Gelatin zymography for the determination of MMP-2 and MMP-9 activities in AATP-treated HT1080 cells. HT1080 cells were treated with AATP (20, 50, and 100 μM) for 1 h and stimulated by PMA (10 ng/mL) for 72 h. Gelatinolytic activities of MMP-2 and MMP-9 in conditioned media were detected by electrophoresis of soluble protein on a gelatin containing 10% polyacrylamide gel. Untreated control was used as a loading control. (**c**) Expression of MMP-2 and MMP-9 in cell Lysates was detected using western blot analysis. β-actin was used as a loading control. HT1080 cells treated with AATP (20, 50, and 100 μM) for 1 h and stimulated by PMA (10 ng/mL) for 24 h. The relative amounts of MMP-2 and MMP-9 were quantified by densitometry measurement (ImageJ). # *p* < 0.001 vs. untreated control, * *p* < 0.05, ** *p* < 0.01 and *** *p* < 0.001 vs. PMA stimulation.

As shown in Figure 3a, PMA stimulation significantly upregulated MMPs RNA expression, whereas AATP treatment efficiently decreased the levels of MMP-1, -2, -3, -9, -13 under PMA stimulation. In zymography analysis and western blotting analysis, we found that AATP treatment significantly suppressed PMA-induced the activities and protein expressions of MMP2 and MMP9 in HT1080 cells in a dose-dependent manner (Figure 3b,c).

### 2.4. AATP Inhibits PMA-induced ERK and JNK Phosphorylation and NF-κB Activation in HT1080 Cells

**Figure 4:**
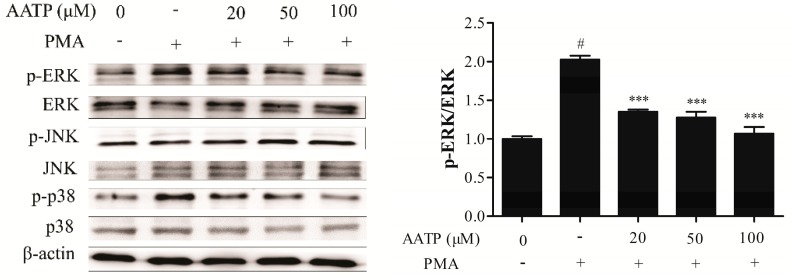

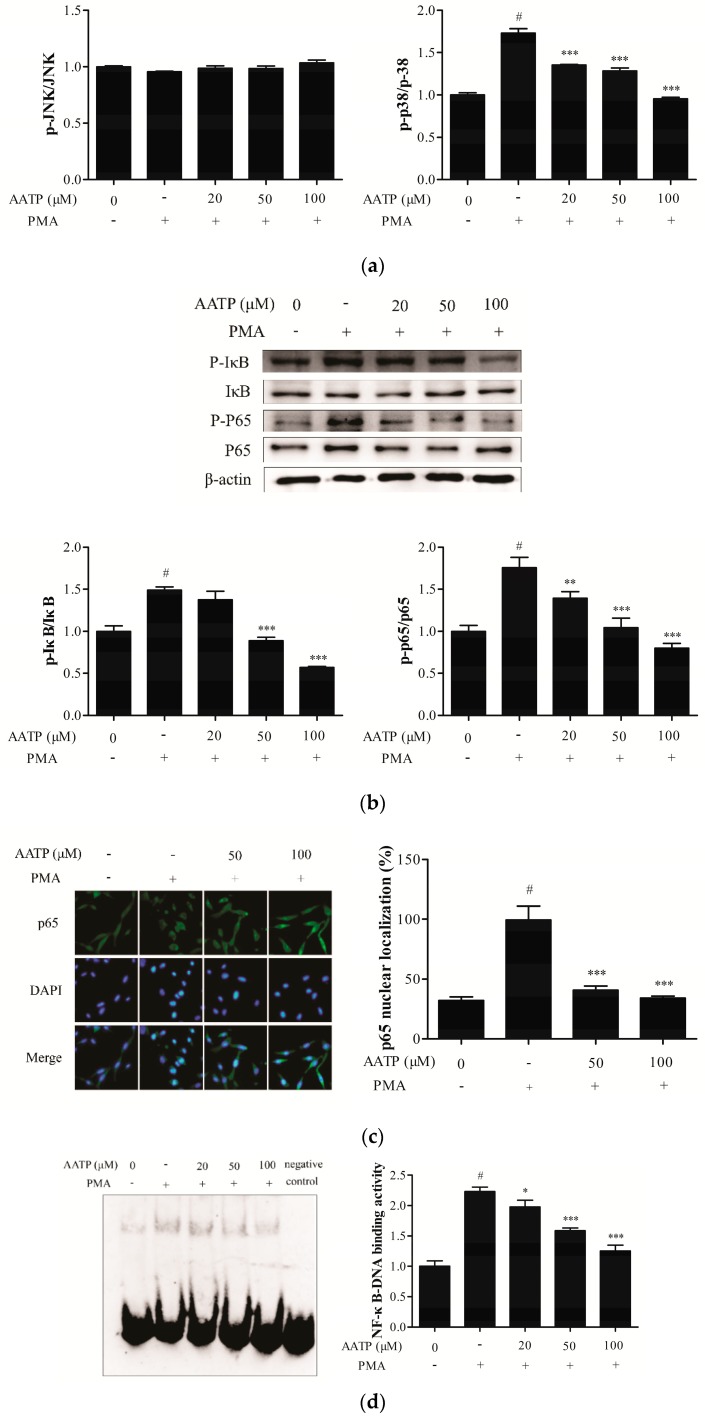
AATP suppressed PMA-induced p-38, ERK, and NF-κB activation in HT1080 cells. After treatment with 20, 50 and 100 μM AATP for 1 h, cells were stimulated with PMA (10 ng/mL) for 24 h. (**a**,**b**) Total cell lysates were evaluated for MAPKs and NF-κB using western blotting. Band intensities were normalized to β-actin expression, and then the relative ratios of phosphorylated form/total form were calculated. (**c**) NF-κB-DNA binding activity was examined by EMSA. Band intensities were normalized to untreated control. (**d**) Nuclear translocation of NF-κBp65 was monitored by an overlay of blue DAPI staining with green p65 immunofluorescence. p-65 nuclear localization was measured. Untreated control was used as a loading control. # *p* < 0.001 vs. untreated control, * *p* < 0.05, ** *p* < 0.01 and *** *p* < 0.001 vs. PMA stimulation.

MAPK and NF-κB signal pathways are related to the expressions of numerous genes that modulate tumor promotion, angiogenesis, metastasis and MMPs expressions. To determine the effect of AATP on MAPK and NF-κB signal pathways in HT1080 cells, the western blotting analysis, p65 translocation and NF-κB activation assay (EMSA) were conducted. As shown in Figure 4a,b, the results of the western blotting assay indicated that AATP treatment markedly suppressed PMA-induced ERK and JNK phosphorylation activation in dose dependent, compared with PMA-induced group. Moreover, the results of p65 translocation and EMSA analysis, the AATP dramatically suppressed p65 nuclear translocation and binding with DNA (Figure 4c,d).

### 2.5. AATP Abolishes VM Formation and Inhibits Secretion of VEGF and Related Protein of Angiogenesis by Suppressing Hypoxia Inducible Factor (HIF)-1α Signal Pathway Under Hypoxic Conditions

**Figure 5:**
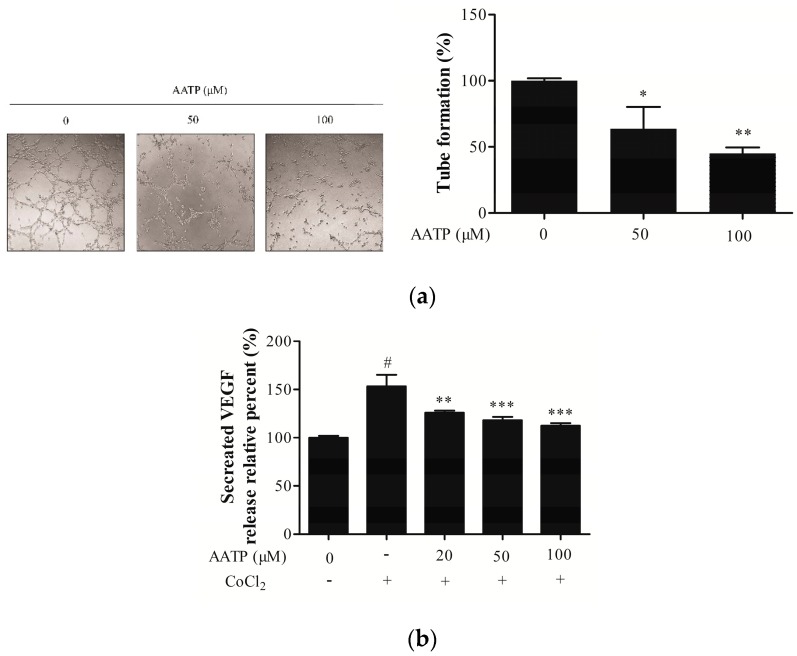

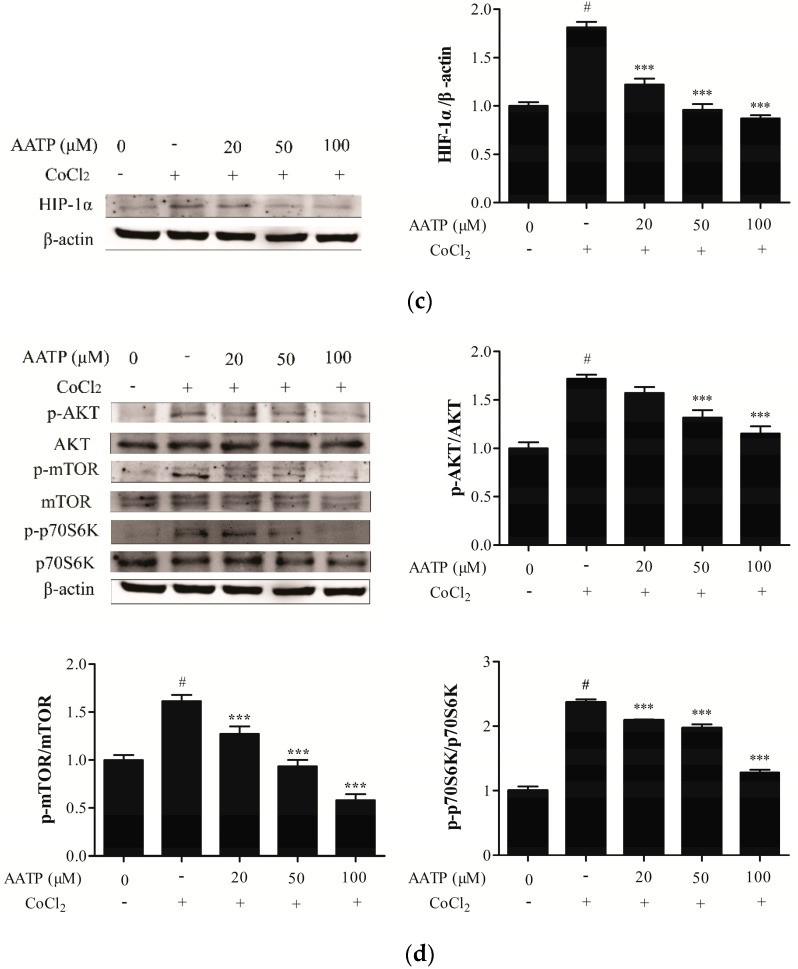
AATP abolishes vasculogenic mimicry (VM) formation and decreases vascular endothelial growth factor (VEGF) secretion in HT1080 cells. (**a**) Cells were seeded on Matrigel pre-coated 96-well plates and incubated in medium containing 50 and 100 μM AATP for 3 h. Then, the photographs of VM formation were taken with an inverted microscope and analyzed by imageJ. (**b**) The cells pre-treated with indicated concentrations AATP for 1 h were stimulated with 100 μM CoCl_2_ for 24 h. The level of VEGF secretion was detected using ELISA kit. AATP inhibits hypoxia-induced expression of HIF-1α and blocks AKT/mTOR/p70S6K signaling in HT1080 cells. (**c**, **d**) Cells were incubated with 20, 50 and 100 μM AATP for 1 h and stimulated with 100 μM CoCl_2_ for 24 h. The expression of HIF-1α, p-AKT/AKT, p-mTOR/mTOR and p-p70S6K/p70S6K were determined by western blotting and β-actin was used as loading controls. # *p* < 0.001 vs. untreated control, * *p* < 0.05, ** *p* < 0.01 and *** *p* < 0.001 vs. CoCl_2_ stimulation.

The rapid growth and metastasis of tumor cells need adequate nutrition and oxygen. Therefore, VM is necessary for tumor cells’ survival, invasion and metastasis. VM formation analysis was employed to investigate the anti-angiogenesis effect of AATP on HT1080 cells. The result showed that VM formation by HT1080 cells on the Matrigel pre-coated wells was abolished through treatment with AATP, as shown in the Figure 5a. VEGF, a pro-angiogenesis protein, is able to promote tumor angiogenesis via stimulating vascular endothelial cells and tumor cells. The level of VEGF secreted by the tumor cell into the medium was determined by ELISA. The ELISA results showed that AATP dose-dependently inhibits the secretion of VEGF from cancer cells (Figure 5b). VEGF is a downstream target of HIF-1α. Therefore, we detected expressions of HIF-1α and AKT/mTOR signal pathway, which is related to angiogenesis. AATP treatment effectively inhibits expression of HIF-1α via blocking AKT/mTOR/p70S6K signaling in a concentration-dependent manner, thus revealing that AATP treatment down-regulated the activation of a pro-angiogenesis factor by suppression of the HIF-1α signal pathway (Figure 5c,d).

### 2.6. Molecular Docking Analysis

**Figure 6:**
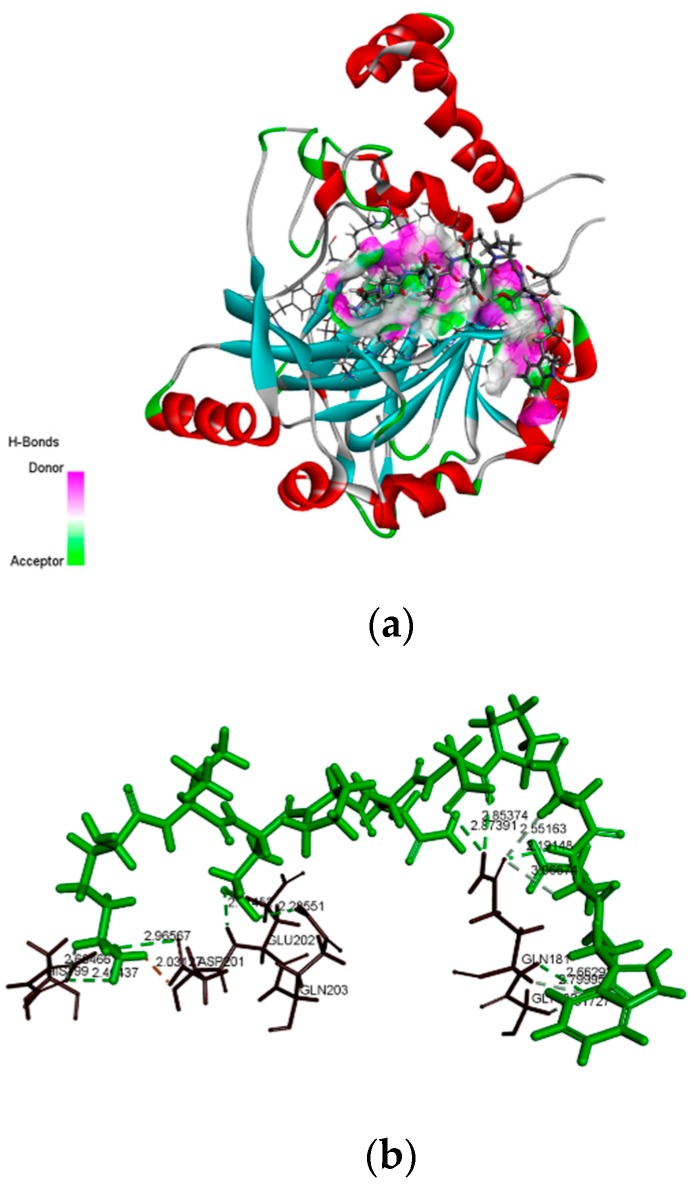
Interaction of the AATP with HIF-1α active site. (**a**) Three dimensional representation of Ligand-1H2L hydrogen bonding. (**b**) Two dimensional representation of Ligand-1H2L hydrogen bonding.

HIF-1α plays an important role in the survival, growth and metastasis of tumor cells, suggesting that HIF-1α inhibitors possess effective effect for tumor treatment. Therefore, we investigated the potential of AATP against HIF-1α and binding affinity using molecular docking approach. As depicted in the Figure 6, AATP combined amino acid residues GLY180, GLN181, HIS199, APS201, GLU202 and GLN203 of HIF-1α, and the strong interaction was supported by the formation of a hydrogen bond, and its docking score was -100.21 kcal/mol. The high binding energies between AATP and HIF-1α contribute to suppression of activity of HIF-1α, resulting in downregulation of downstream reactions that relate to tumor metastasis and VM formation.

## 3. Discussion

In the present study, we investigated the effect of a polypeptide AATP isolated from abalone on tumor metastasis and VM formation in HT1080 cells. AATP can significantly suppress tumor cells metastasis (Figure 2a,b) and VM formation (Figure 5a), which are essential steps in tumor progression. Previous studies have been found that abalone visceral extracts showed remarkable inhibitory effect on tumor progression by regulating the expressions of tumorigenic factors, such as Cox-2, EGF, VEGF and FGF [21]. It is suggested that the extract obtained from the glycoprotein of *H.discus hannai* possess effective effect on anti-cancer by the host’s responses [22], and investigations revealed that water extract from abalone was a source of bioactive molecule against many types of tumors [23]. Currently, the activity of peptides has been paid more and more attention to by researchers. Peptides have advantages in shape to agonist or antagonist bind sites of the receptor, because peptides can bind to a protein receptor and have few off-target effects [24]. Moreover, peptides are applicable as lead compounds for pharmacophores or the design of drug-like molecules with incorporated secondary structural elements [25,26]. Up to now, many marine-derived peptides, such as ziconotide, brentuximab vedotin, kahalalide F, and glembatumumab vedotin, have been used successfully in clinical trials and in the market [24]. 

The generation of blood vessels is pivotal for tumor cells survival, growth and metastasis. Besides traditional tumor angiogenesis and vasculogenesis, VM, a functional vascular channel developed by tumor cells, is also responsible for the tumor growing and tumor initiation [27]. It can provide nutrition to tumor cells and allow tumor cells to grow through them when the blood vessels by endothelial cells are insufficient to the growth of tumor tissue [28]. Hypoxia within the tumor microenvironment serves as an important causative factor for VM formation because it can increase the generation of pro-angiogenic factors, such as VEGF and MMPs, which facilitate the formation blood vessel and the splitting of pre-existing vessels, respectively [29,30,31]. Therefore, VM plays a critical role in blood supply in malignant tumors [32], and targeting VM may provide a promising strategy to regulate the spread of tumors. 

In this study, under hypoxic conditions, AATP treatment dramatically suppressed expression of HIF-1α induced by hypoxia, and blocked the AKT/mTOR/p70S6K signal pathway related to angiogenesis (Figure 5c,d), which lead to deregulation of VEGF (Figure 5b). Kim [33] found ELH can attenuate HIF-1α accumulation by blocking phosphorylation of AKT/mTOR/p70S6K to inhibit tumor angiogenesis. It has been reported that VEGF can induce cell proliferation, metastasis and tube formation [34], and decreased expression of VEGF lead to suppress angiogenesis in MDA-MB-435 cells [35]. Moreover, AATP markedly downregulated tumor cells metastasis, including migration, invasion and activity of MMPs by MAPKs (p38 and ERK) and NF-κB (p65 and IκB) signaling (Figure 4). It was reported that MAPKs and NF-κB have relationships with the expressions of target genes associated with tumor promotion, angiogenesis, metastasis and MMPs [33,34,35,36,37,38]. Lu [39] found that emodin could effectively inhibit the anti-inflammatory through blocking NF-κB activation and MAPKs pathway on PMA plus A23187-stimulated BMMCs. Cao [40] exhibited that ginkgetin suppresses growth of breast carcinoma by regulating the MAPKs pathway. And fucoxanthin extract inhibits MMPs by regulating NF-κB and MAPKs pathways in human fibrosarcoma cells [41]. This suggests that regulation of NF-κB and MAPKs pathways are closely related to activity and expression of MMP-2 and MMP-9.

Molecular docking is based on spatial matching and energy matching, simulating the binding ability between ligands and human receptors, and the docking result showed that AATP can combine with GLY180, GLN181, HIS199, APS201, GLU202 and GLN203 of the active site of HIF-1α, leading to the suppression of HIF-1α activity (Figure 6). This suggested that peptide AATP and receptors HIF-1α have a similar key and lock recognition relationship in the configuration, resulting that AATP binds to the active site of the receptor and occupies the spatial position of HIF-1α. Therefore, HIF-1α failed to bind the hypoxia response element of the initiator, leading to downregulation of target genes relevant to tumor metastasis and VM formation. Additionally, the amino acid composition of peptides is responsible for its bioactivity. In the Lys-Val-Asp-Ala-Gln-Asp-Pro-Ser-Glu-Trp (AATP), the amino acids Glu, Asp, Pro and Lys could effectively inhibit activity of MMP-2 and MMPs [42]. In particular, the amino acids Trp, Tyr, Met, Gly, Cys, His, Val and Pro in a peptide can markedly elevate the bioactivity of the peptide and hydrophobic acid residues Val and Pro contribute to the formation of oil-water interfaces, leading to the scavenging of free radicals from the lipid phase [43,44]. Huang found that a novel tripeptide (Gln-Pro-Lys) derived from the sepia ink possesses anti-tumor properties in DU-145 cells [45]. 

In summary, we demonstrated that AATP isolated from abalone (*Haliotis discus hannai*) suppresses the metastasis and VM formation on HT1080 cells via downregulating MMPs and VEGF. In addition, the result of molecular docking showed that AATP combines with HIF-1α via a hydrogen bond, resulting in suppression of HIF-1α activity, which was accordant with the result of western blotting. Therefore, all results in vitro revealed that AATP can effectively inhibit tumor cells metastasis and VM formation, which provides the basis for the further application of AATP to animal experiments. Furthermore, with regard to the AATP therapeutical setting, there are limitations like most peptides, including delivery, short half-life and orally available as well as clear from kidneys after intravenous administration, which needs to be overcome by using different design strategies in the future.

## 4. Materials and Methods 

### 4.1. Chemicals and Reagents

Human fibrosarcoma cells (HT1080 cell) and human umbilical vein endothelial cells (HUVEC) were provided by Guangzhou Cellcook Biotech Co., Ltd. (Guangzhou, China). Dulbecco’s modified Eagle’s minimal essential medium (DMEM) and penicillin/streptomycin were purchased from Gibco (Grand Island, NY, USA). Fetal bovine serum (FBS) was from Vigonob (UY). 3-(4, 5-dimethyl-thiazol-2-yl)-2, 5-diphenyltetrazoliumbromide (MTT) were obtained from Shanghai Aladdin Bio-Chem Technology Co., Ltd. (Shanghai, China). Antibody against p65, p-p65, IκB, p-IκB, ERK, p-ERK, p-38, p-p38, JNK, p-JNK, β-actin, MMP2 and MMP9 were provided by Santa Cruz Biotechnology (Santa Cruz, CA, USA). Antibody HIF-1α, AKT, p-AKT, p-mTOR, mTOR, p-p70S6K, p70S6K and horse anti-mouse IgG were purchased from Cell Signaling Technology (Boston, MA, USA). Matrigel was from BD Biosciences (San Jose, CA, USA). Phorbol 12-myristate 13-acetate (PMA) and CoCl_2_ were provided by Sigma-Aldrich (St. Louis, MO, USA). The isolated peptide AATP (MW = 1214.30 Da) was from our studies previously [20]. 

### 4.2. Cell Viability Assay

HT1080 cells and HUVECs were cultured in 96-well plate in growth medium for 24 h. Then fresh media containing different concentrations (10, 20, 50, and 100 μM) of AATP were added. After 24 h, 100 μL MTT (1 mg/mL) was added into each well for 4 h. Then, adding 100 μL DMSO to dissolve formazan crystals, and the absorbance was measured at 540 nm. 

### 4.3. Colony Formation Assay

HT1080 cells were placed in 6-well plate (500 cells/well) in DMEM containing 10% serum. After 24 h, the medium was replaced with fresh medium containing different concentrations (20, 50 and 100 μM) of AATP, and cultured for 7 days. The colonies were stained with 0.2% crystal violet/methanol (*w*/*v*) solution for 20 min at room temperature, washed with distilled water and then photographed. 

### 4.4. Cells Migration Assay

The cell migration ability was estimated by injury healing assay. Briefly, HT1080 cells were seeded in a 24-well plate. The cells were scratched using a sterile pipette tip, and then washed with PBS to remove cell debris. Cells were treated with various concentrations of AATP (10, 20, 50 and 100 μM). And the cell migration across injury line was observed using a microscope (JiDi, GD30, Guangzhou, China) and recorded photographically at 0, 12 and 24 h, respectively.

### 4.5. Cancer Cell Spheroid Invasion Assay

To investigate the inhibitory effect of AATP on cell invasion, the cancer cell spheroid invasion assay was performed to simulate the internal environment and assess invasion in a 3-dimensional (3D) setting. Briefly, single-cell suspension (1 × 10^5^ cells/mL) was seeded in the lid of the dish for 72 h. Next, the spheroids are pooled, and 80 μL cell spheroids were added in 400 μL mixture of Matrigel and type I collagen at 4 °C, plated in 48-well plates and incubated at 37 °C to solidify into a 3D culture. After 30 min, warm media with the indicated concentrations (50 and 100 μM) of AATP was added. The result of cell invasion was observed using a microscope, and recorded at 0, 24, and 48 h.

### 4.6. RNA Extraction and Quantitative Real Time PCR (qPCR)

Total RNA was extracted and purified using a Nucleic acid purification kit (DSBIO, Guangzhou, China). The purified RNA (1 μg) was reverse transcribed to synthesize cDNA for RT-PCR using the HiScript II 1st Strand cDNA Synthesis Kit (+gDNA wiper) (Vazyme, Nanjing, China). Quantitative PCR reaction was carried out using CFX96 Real-Time System (BIO-RAD, Hercules, CA, USA), and forward and reverse primers of the target gene (MMP-1, MMP-2, MMP-3, MMP-9 and MMP-13) were shown in Table 1. For quantification of target gene, transcript values were analyzed using the 2^−ΔΔCt^ method and β-actin was considered as the reference.

### 4.7. Gelatinolytic Activity

Activities of MMP-2 and -9 of HT1080 cells were determined by gelatin zymography. Briefly, cells were seeded in 24-well plates with a density of 2 × 10^5^ cells/well and pretreated with different concentrations of AATP (20, 50 and 100 μM) for 1 h, and then stimulated by PMA (10 ng/mL) for 72 h. Cell medium was collected to conduct gel electrophoresis. Finally, areas of gelatin hydrolyzed by MMPs were visualized as clear zones against blue backgrounds by Coomassie Blue staining, and the intensities of the bands were estimated by ImageJ software (National Institute of Mental Health, Bethesda, MD, USA).

### 4.8. Western Blotting 

Cellular protein was harvested and lysed using RIPA buffer with 1% PMSF. And then equivalent amounts of proteins (30 μg) were separated using SDS-PAGE, and subsequently transferred to NC membranes. The membrane was blocked with 5% skim milk at room temperature. After 3 h, the membrane was incubated with primary antibodies and secondary antibodies for 2 h respectively at room temperature. Finally, the membrane using enhanced chemiluminescence (ECL) was visualized by detection system (Syngene, Cambridge, UK).

### 4.9. Immunofluorescence Staining

HT1080 cells treated with AATP (0, 50, and 100 μM) for 1 h were incubated with PMA (10 ng/mL) for 24 h and the medium was discarded. After being fixed with 4% paraformaldehyde (PFA), the cells were permeabilized with 0.2% Triton X-100 for 10 min and washed with PBS thrice. The cells were blocked with 5% BSA at room temperature for 1 h. Then, cells were incubated with diluted anti-p65 antibody overnight at 4 °C, and the primary antibodies were removed. Cells were washed using PBS three times, and incubated with secondary antibody for 2 h. The nuclear was stained with DAPI and images were taken by an inverted fluorescence microscope (Olympus Opticals, Tokyo, Japan).

### 4.10. NF-κB Activation Assay (Electrophoretic Mobility Shift Assays, EMSA)

The nuclear protein was obtained using the nuclear and cytoplasmic protein extraction kit (Beyotime, Shanghai, China). According to instructions, the complexes of protein and DNA and unbound probes were separated by 6% non-denaturing polyacrylamide gel electrophoresis, and subsequently transferred to Nylon membranes. Membrane was crosslinked for 15 min under UV light and blocked with blocking solution containing streptavidin-HRP conjugate. Then the membrane was balanced with a detection balance solution for 15 min and visualized with an enhanced chemiluminescence (ECL) detection system.

### 4.11. Assaying the Release of VEGF

Cells were plated at the same density in 6-well plates and treated with various concentrations (20, 50, and 100 μM) of AATP for 1 h and were subsequently stimulated with CoCl_2_ (100 μM). After 24 h, conditional media were collected, and the quantity of VEGF was analyzed using Elisa Kit (Neobioscience, Shanghai, China).

### 4.12. Molecular Docking Analysis

The three-dimensional structures of HIF-1α (PDB ID: 1H2L) were obtained from the Protein Data Bank archive (PDB). The structure of AATP and HIF-1α were prepared by Discovery Studio 3.5 software. Molecular docking of AATP and HIF-1α protein binding sites was performed by using CDOCKER protocol of DS 3.5. The small molecule conformation was searched by high temperature dynamics method, and they were optimized in the active sites area of the acceptor by simulated annealing.

### 4.13. Statistical Analysis

The data were presented as mean ± SD (*n* = 3) and all results were analyzed using the GraphPad Prism 5 software (San Diego, CA, USA). Date was analyzed by one-way ANOVA in the figures, and *p* value < 0.05 was considered statistically significant.

## Figures and Tables

**Table 1:** Details of primers for quantification of RT-PCR detection.

Primer ID	Primer Sequence (5′→3′)
MMP-1	F: 5′-AGCCATCACTTACCTTGCACTGAG-3′
	R: 5′-RCCACATCAGGCACTCCACATCTG-3′
MMP-2	F: 5′-AGCCAAGCGGTCTAAGTCCAGAG-3′
	R: 5′-GGAATGAAGCACAGCAGGTCTCAG-3′
MMP-3	F: 5′-ACGCACAGCAACAGTAGGATTGG-3′
	R: 5′-GAGGCAGGCAAGACAGCAAGG-3′
MMP-9	F: 5′-TCCTGGTGCTCCTGGTGCTG-3′
	R: 5′-CTGCCTGTCGGTGAGATTGGTTC-3′
MMP-13	F: 5′-AGTCATGGAGCTTGCTGCATTCTC-3′
	R: 5′-TCCTGGCTGCCTTCCTCTTCTTG-3′
β-actin	F: 5′-CCTGGCACCCAGCACAAT-3′
	R: 5′-GGGCCGGACTCGTCATAC-3′
